# Gamified IMU Sensor-Based System for Training Head-Orientation Tasks in Healthy Teenagers: Event-Driven Architecture and Randomised Pilot Feasibility Study

**DOI:** 10.3390/s26196315

**Published:** 2026-10-06

**Authors:** Andrea Calleja-Caballero, Héctor Sánchez San-Blas, Josué Prieto Prieto, Fátima Pérez-Robledo, André Filipe Sales Mendes

**Affiliations:** 1 Department of Nursing and Physiotherapy, University of Salamanca, 37007 Salamanca, Spain; andreacallejac@usal.es; 2 Neurorehabilitation Research Group, Institute of Biomedical Research of Salamanca (IBSAL), 37007 Salamanca, Spain; 3 Ontology Engineering Group, ETSI Informáticos, Polytechnic University of Madrid, 28223 Madrid, Spain; h.sanchezsanblas@upm.es; 4 Instituto Universitario de Ciencias de la Educación (IUCE), University of Salamanca, 37008 Salamanca, Spain; 5 Department of Computer Science and Automation, University of Salamanca, 37008 Salamanca, Spain; 6 CREA-Salud Research Group, Salamanca Primary Care Management, Institute of Biomedical Research of Salamanca (IBSAL), 37007 Salamanca, Spain

**Keywords:** BLE sensors, event-driven architecture, edge computing, gamification, human–computer interaction, head orientation

## Abstract

Sensor systems integrated with gamified environments have increasingly been incorporated into the monitoring of musculoskeletal disorders. This study evaluates the operational feasibility of a motion-capture system based on an inertial measurement unit for training head-orientation tasks. Because a single sensor is placed on the head without thoracic fixation, the system estimates head orientation relative to space rather than isolated cervical motion. The event-driven architecture is organised into five logical layers and uses an asynchronous publish–subscribe bus for data acquisition, sensor fusion through the Madgwick filter, and local rendering in WebGL. A randomised pilot study involving 22 teenagers allocated to two parallel groups (11 per group) was conducted over eight weeks (three sessions per week). Participants were assigned to either the gamified system or a comparable active cervical exercise program. No participant-reported connectivity or hardware events prevented session completion. Self-reported adherence was 100%, and no adverse events were reported. Exploratory analyses showed that both groups reduced task completion time, whereas the reduction in time spent outside the target trajectory was statistically significant only in the experimental group; the between-group comparison of pre–post changes was not statistically significant. These findings indicate the system’s operational feasibility in healthy teenagers.

## 1. Introduction

Neck pain is one of the most common musculoskeletal disorders and a relevant cause of functional limitation [1,2]. Its prevalence among teenagers has increased in recent decades [3,4], with potential consequences for quality of life and symptom persistence into adulthood [5,6]. Sedentary behaviour and prolonged use of electronic devices have been associated with this condition [7]. Continuous screen exposure may contribute to postural changes and increase mechanical loading on the cervical spine [8,9].

From a biomechanical perspective, neck pain alters proprioception and the coordination of the deep cervical musculature [10,11], thereby impairing stability and sensorimotor control [12]. Therefore, accurate assessment and early rehabilitation of motor control are essential [13,14]. However, conventional clinical practice has methodological limitations: assessments are often qualitative, and rehabilitation programs have high dropout rates because young patients often lack motivation.

To address these limitations, researchers have developed sensor-based tools. This study aims to evaluate the operational feasibility of an *e-Health* system based on wearable inertial sensors (*wearables*) and gamified audiovisual feedback. The system uses a modular, event-driven software architecture to manage concurrent processing of biomedical signals and graphical interfaces. Its exploratory application to training head-orientation tasks was evaluated in a cohort of healthy teenagers.

The architecture performs inertial data acquisition, sensor fusion, and interactive rendering directly in the browser, without relying on remote servers within the feedback loop. Kinematic events are communicated to independent modules, allowing modification of either the sensor or the graphics engine without altering the evaluation logic. The eight-week randomised pilot study in healthy teenagers provides preliminary data on operational feasibility and useful estimates for planning future trials.

The remainder of this article is structured as follows: Section 2 presents the State of the Art, with the evolution of motion-capture technologies, the role of audiovisual *feedback*, and processing architectures. Section 3 details the design of the proposed architecture and describes its modular layers, including the benchtop technical verification of latency, packet loss, and frame rate (Section 3.7). Section 4 describes the methodology of the pilot feasibility study conducted in a teenage population. Section 5 presents the technical feasibility results and the exploratory kinematic metrics obtained. Finally, Section 6 presents the discussion, and Section 7 summarises the conclusions and future research directions.

## 2. Background

### 2.1. Cervical Motion-Capture Technologies and Clinical Biomechanics

The current literature on digital biometric technologies establishes the V3 framework for their validation: hardware Verification, Analytical Validation of the algorithm, and Clinical Validation of the construct [15]. In this context, the study of the cervical spine represents a substantial biomechanical challenge due to its complex functional anatomy (C1–C7) and its high mobility across the three rotational degrees of freedom (pitch, roll, and yaw) [16]. Historically, clinicians have assessed cervical motor control using mechanical tools such as analogue goniometers, inclinometers, and static qualitative observational tests [17]. Although these traditional methods are useful for a macroscopic assessment of range of motion (ROM), they lack the sensitivity and dynamic resolution required to detect subtle alterations in proprioception and fine motor control and are also highly susceptible to inter-rater bias [14].

To overcome these limitations of conventional analogue methods, research settings have long relied on optoelectronic motion-capture systems based on multiple infrared cameras and retroreflective markers, such as Vicon or Qualisys systems. Although widely regarded as the *gold standard* for three-dimensional geometric accuracy, these systems have inherent logistical and economic disadvantages that hinder widespread adoption: they require dedicated biomechanics laboratories, involve high acquisition and maintenance costs, and depend on complex calibration procedures. In addition, they are susceptible to marker occlusion during dynamic exercises, limiting their applicability in routine clinical practice and telerehabilitation settings.

As a viable alternative, advances in microelectromechanical systems (MEMS) miniaturisation have promoted the use of wearable Inertial Measurement Units (IMUs). IMUs, which comprise triaxial accelerometers, gyroscopes, and often magnetometers, have demonstrated accuracy comparable to that of optical systems [18]. Because accelerometers are susceptible to high-frequency noise during abrupt movements and gyroscopes are affected by cumulative orientation drift (*drift*) over time, the current literature emphasises the critical importance of sensor-fusion algorithms. Filters such as the Extended Kalman Filter (EKF), the Madgwick algorithm, and Mahony’s nonlinear complementary filter [19] jointly process inertial signals, compensate for each sensor’s errors, and produce stable three-dimensional orientations (quaternions) in real time. Recent studies have rigorously validated inertial technologies against optoelectronic systems for the cervical region [18]. However, the accuracy of these measurements depends on the physical sensor configuration: accurately measuring cervical kinematics ideally requires assessment of the relative motion between the head and thorax (using two sensors) or strict stabilisation of the trunk, since the use of a single frontal IMU may capture unintended thoracic compensations [18].

### 2.2. HCI, Gamification, and Audiovisual Feedback in Rehabilitation

Active therapeutic exercise is a conservative intervention used to improve function, reduce pain, and increase cervical ROM [20,21]. Its effectiveness in practice depends, among other factors, on adherence. In pediatric and teenage populations, the repetitive nature of postural exercises may reduce participation in the program [22]. For this reason, researchers have investigated Human–Computer Interaction (HCI) approaches and serious games (*exergames* or *serious games*) as tools to support therapeutic exercise [23].

Gamification can incorporate goals, progress information, and audiovisual feedback into exercise. According to classical motor-learning models, *feedback* plays an important role during the initial stages of skill acquisition [24]. Some studies have linked interfaces that provide immediate audiovisual feedback to greater engagement and perceived effort [25,26]. However, the intensity and timing of *feedback* should match the task: continuous exposure may promote dependence on external cues and impair long-term retention [27,28]. Recent reviews of corrective *feedback* based on visual signals for motor-skill training agree that signal design (modality, frequency, and timing) influences its effectiveness [29]. Therefore, the platform allows configuration of the modality and intensity of *feedback*. In teenagers, virtual environments and gamification have been associated with favourable outcomes in motor skills and perceived effort, although this does not in itself demonstrate clinical efficacy [26].

Evidence on the clinical superiority of gamification remains heterogeneous. Some trials have reported benefits in adherence and satisfaction, whereas systematic reviews and meta-analyses of virtual reality and serious games in individuals with neck pain [30,31,32] have found inconsistent results. Improvements in motivation and, in some studies, short-term ROM do not consistently demonstrate an advantage over conventional physiotherapy for chronic pain. Therefore, researchers should first establish the technical and methodological feasibility of these platforms before evaluating hypotheses of clinical efficacy.

End-to-end latency is a relevant aspect of a feedback interface. In closed-loop interaction environments, delay between movement and visual updating may affect performance and perception, although no single threshold applies to all tasks. Waltemate et al. [33] observed effects on motor performance with delays on the order of tens of milliseconds, and reviews on motor learning emphasise the importance of temporal proximity between action and *feedback* [34]. A value of 100 ms is often used as a design guideline in interactive applications, but it does not constitute a validated limit for cervical rehabilitation. High latency may impair the user experience and hinder the association between corrective *feedback* and the movement that triggers it.

Meeting these timing constraints represents a substantial computational challenge, particularly when processing high-frequency inertial signals. The system must simultaneously and continuously acquire raw data through wireless protocols such as Bluetooth Low Energy (BLE), execute computationally intensive sensor-fusion algorithms (including direction cosine matrix and quaternion calculations), compute two- or three-dimensional spatial collision logic, issue multimedia alerts, and insert structured data into a database for clinical monitoring. The literature on body sensor networks identifies managing concurrency and computational resources as an open challenge in this type of platform [35]. When these subtasks are handled through tightly coupled designs executed synchronously within a single thread, any fluctuation (*jitter*) in BLE reception can affect graphical-interface rendering and, consequently, perceived latency. This relationship is presented here as a design motivation derived from the execution model, rather than as an empirically established finding for specific e-Health systems.

To mitigate these system-level bottlenecks, this work implements an event-driven system that runs natively in the user’s web browser rather than offloading processing to the cloud. This work presents an initial empirical assessment of operational feasibility through a pilot study conducted with a cohort of healthy teenagers.

## 3. Event-Driven Modular Architecture

The proposed system uses a modular, event-driven architecture designed to support real-time acquisition, processing, visualisation, and analysis of head-orientation data. The architecture separates the main computational responsibilities into five logical layers: (1) the physical layer, (2) the edge-processing layer (*edge*), (3) the asynchronous event bus, (4) the application layer, and (5) the portal and management layer. This separation reduces coupling between modules and allows sensor acquisition, kinematic processing, graphical rendering, performance evaluation, and clinical supervision to operate independently.

**Figure 1:**
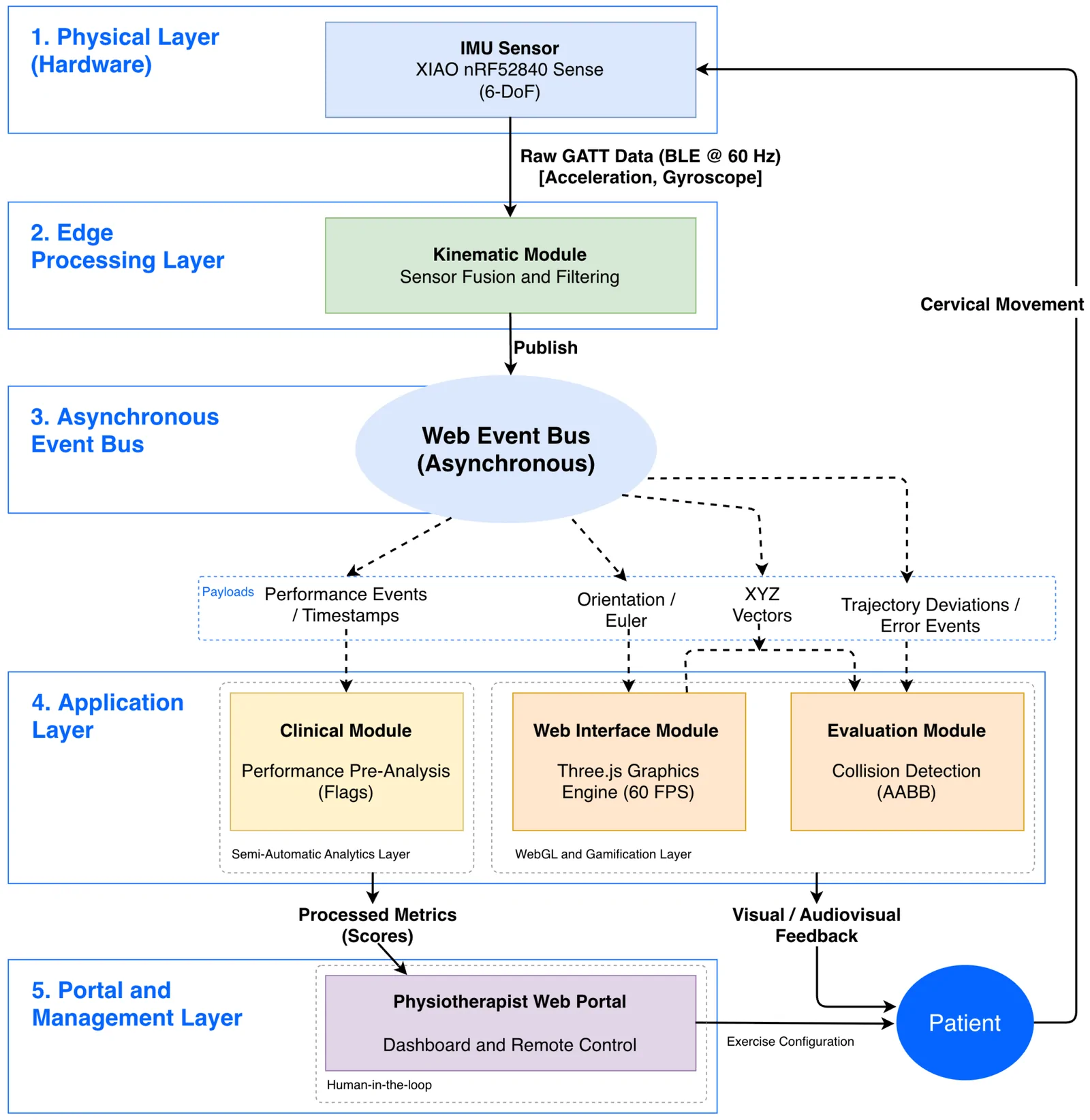
Modular event-driven architecture of the proposed system. Solid arrows represent the main processing flow and dashed arrows the asynchronous distribution through the web event bus. Inertial data are processed in the edge layer and published as specific events (orientation, spatial, performance) for each consuming module, supporting real-time processing and subsequent clinical supervision.

Figure 1 summarises the main system components and the information exchanged among them. Solid arrows represent the main sequential flow of acquisition, processing, *feedback* generation, and exercise configuration. Dashed arrows represent the asynchronous distribution of the different event types published through the web bus. The central component is an asynchronous web event bus that decouples information producers and consumers, thereby avoiding the need to establish direct point-to-point communication between modules.

This event-driven approach is particularly relevant to interactive motor-training applications because multiple tasks must be executed concurrently. The system must continuously receive inertial measurements, estimate orientation, update the virtual environment, detect trajectory errors, calculate performance metrics, and generate visual *feedback*. Decoupling these operations prevents temporary delays in one module from unnecessarily blocking the rest of the processing pipeline.

### 3.1. Physical Layer: Wearable IMU Sensor

**Figure 2:**
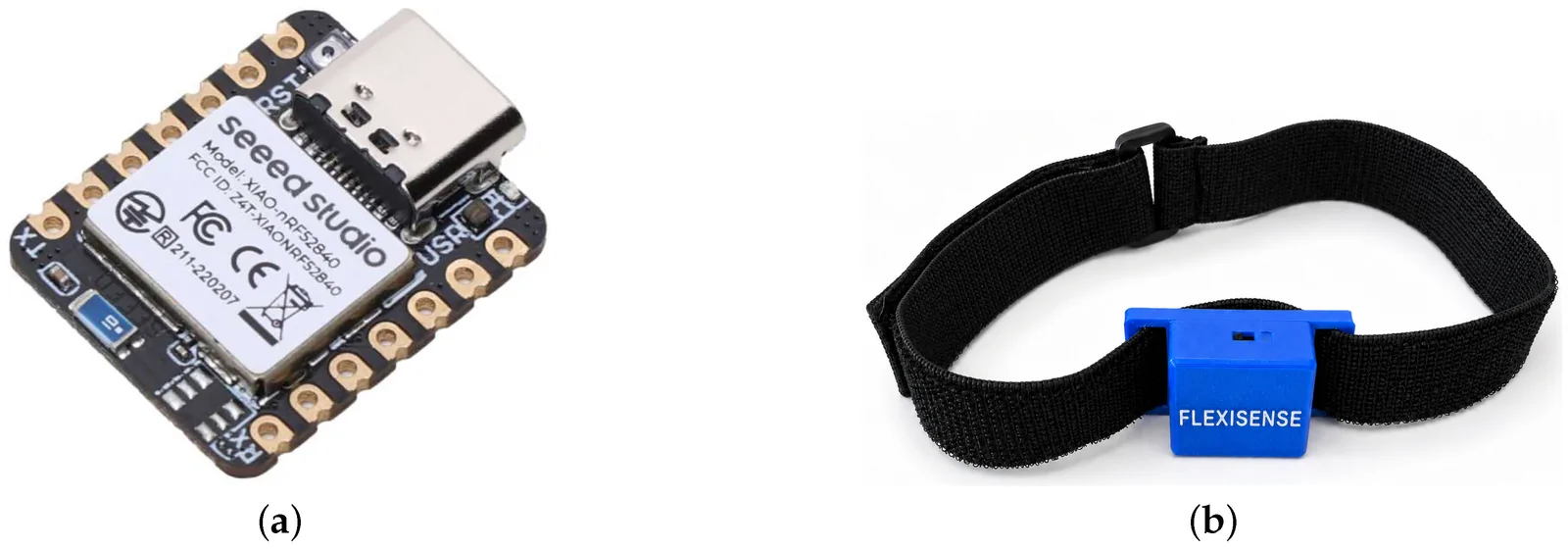
Wearable device used in the system. (**a**) Seeed Studio XIAO nRF52840 Sense development board. (**b**) Assembled device in its protective enclosure (Flexy Sense) with an adjustable head strap.

The physical layer contains the body-worn sensing device (*wearable*) used to capture the participant’s head movement. The system uses a Seeed Studio XIAO nRF52840 Sense board equipped with a six-degree-of-freedom inertial measurement unit (IMU). The IMU integrates a three-axis accelerometer and a three-axis gyroscope, measuring linear acceleration and angular velocity along the three spatial axes. The inertial unit integrated into this board is an STMicroelectronics LSM6DS3TR-C, whose complete specifications (selectable measurement ranges, 16-bit resolution per axis, and noise characteristics) are available in the manufacturer’s documentation. In the present implementation, the sensor was configured with a nominal sampling frequency of 60 Hz, and its data were transmitted through a custom GATT service over BLE. Figure 2 shows the board used and the assembled device housed in its enclosure with the head-mounted fastening strap.

**Figure 3:**
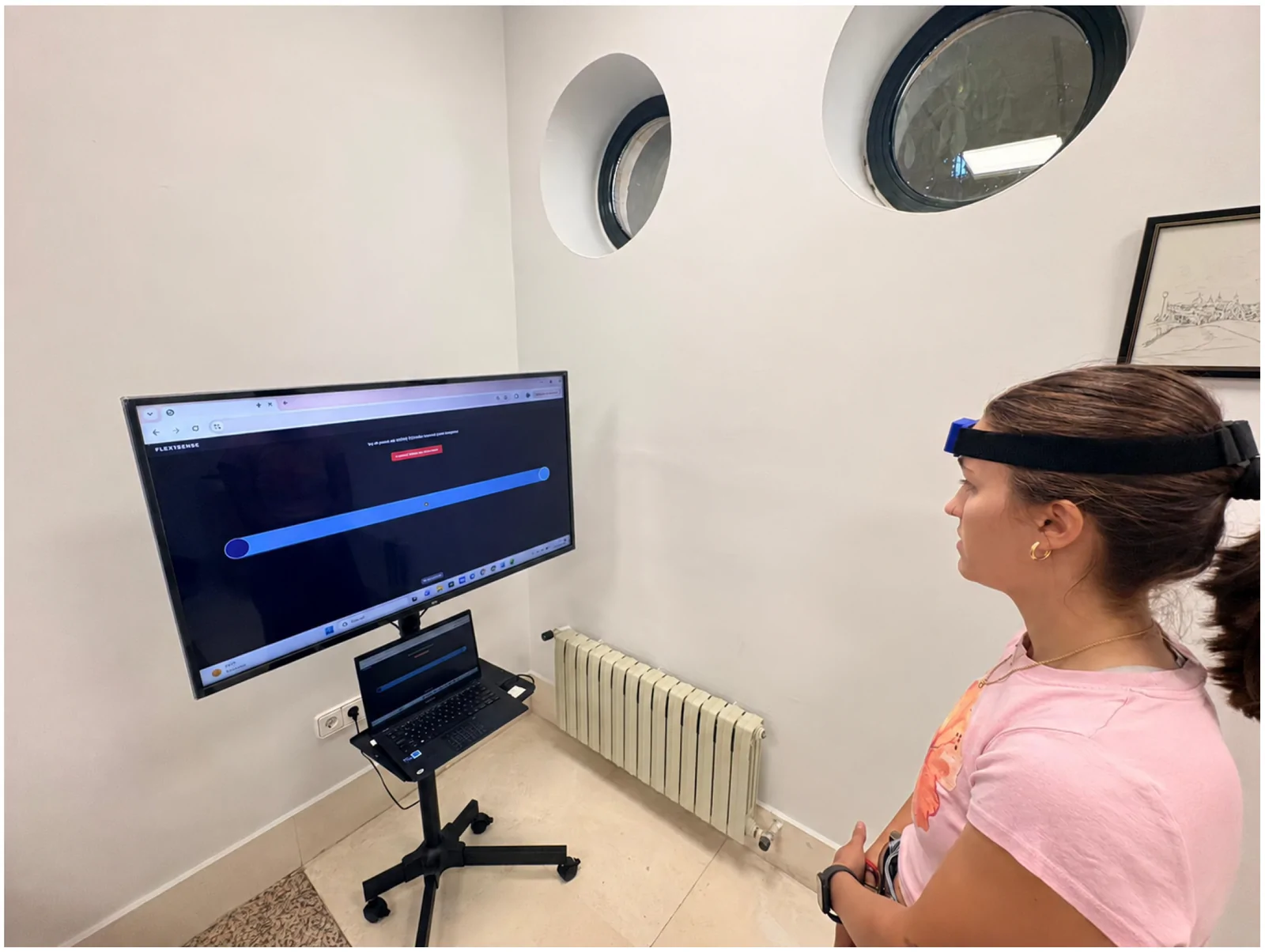
Experimental setup during a training session. The wearable device is secured to the participant’s head with an adjustable strap, and the participant stands approximately 2 m away from the screen while following the moving target with head movements.

Figure 3 shows the experimental setup during a training session, with the device positioned on the participant’s head and the display located approximately 2 m away.

The sensor is positioned on the participant’s head to capture the movements performed during the training tasks. The embedded *firmware* periodically reads the accelerometer and gyroscope registers and packages the resulting values into structured data frames.

The sensor transmits measurements wirelessly via Bluetooth Low Energy (BLE). A custom service sends the raw inertial data to the browser-based application at an approximate sampling frequency of 60 Hz. Each transmitted sample includes the acceleration and angular velocity components, together with a *timestamp* used to preserve the temporal order of the measurements.

The physical layer is deliberately limited to data acquisition and communication. The edge-processing layer handles computationally intensive operations, including sensor fusion and orientation estimation. This distribution reduces the processing requirements of the *wearable* device and facilitates future replacement or upgrading of the sensing *hardware*.

### 3.2. Edge-Processing Layer: Kinematic Module

The *edge*-processing layer transforms raw inertial measurements into meaningful kinematic information. Its main component is the kinematic module, which receives the acceleration and gyroscope samples transmitted by the *wearable* sensor.

Initially, the module performs basic signal conditioning, including validating the received data frames, temporally ordering the samples, removing incomplete measurements, and normalising the sensor values. Sensor calibration parameters can also compensate for static deviations (*offsets*) and measurement bias.

Following preprocessing, the system employs a sensor-fusion algorithm based on the Madgwick filter [36] to combine accelerometer and gyroscope information. The accelerometer provides a gravity-based reference that contributes to tilt estimation, whereas the gyroscope captures rapid angular changes but is susceptible to cumulative drift (*drift*). Mathematically, the orientation estimate in quaternion form, $q_{t}$, is updated at each time step by minimising the alignment error. The basic update equation can be expressed as:

(1) $$q_{t}=q_{t-1}+{\dot{q}}_{\omega ,t}\Delta t-\beta \frac{\nabla f}{∥\nabla f∥}$$

where ${\dot{q}}_{\omega ,t}$ is the rate of change of orientation derived from the gyroscope, β is the gain factor that determines the weighting of the accelerometer contribution, and ∇f represents the gradient of the cost function associated with the gravitational alignment error. This formulation has a low computational cost, making it suitable for execution on *edge* devices.

Internally, the system represents orientation using quaternions. Quaternion-based processing avoids the singularities associated with direct Euler-angle calculations (*gimbal lock*) and provides a compact representation suitable for continuous three-dimensional rotation. When required by the graphical interface or analytical modules, the quaternion is converted into Euler angles corresponding to pitch (*pitch*), roll (*roll*), and yaw (*yaw*).

The processing results are published to the asynchronous web event bus. Each kinematic event may include the estimated quaternion, its Euler-angle representation, the corresponding spatial vectors, and the associated timestamp. Based on this information, the bus generates and distributes specific *payloads* tailored to the requirements of the different consumer modules.

All processing is performed locally on the user’s device. Consequently, the real-time interaction loop does not depend on continuous communication with a remote server. This *edge-computing* strategy reduces network dependence and is intended to minimise the delay between physical movement and visual feedback.

### 3.3. Asynchronous Web Event Bus

The asynchronous web event bus serves as the architecture’s communication core. It implements a publish–subscribe interaction model in which *software* modules publish events without knowing which components will consume them. Other modules subscribe only to the event categories they need to operate.

This design replaces tightly coupled calls between modules with asynchronous message exchange. The kinematic module publishes processed information to the web bus, which distributes it through specialised *payloads* according to each consumer’s needs. The WebGL interface receives orientation data as quaternions or Euler angles; the evaluation module receives XYZ vectors and events related to trajectory deviations or errors; and the clinical module consumes aggregated performance events with corresponding timestamps. This specialisation prevents each component from processing information it does not need for its function.

The *payloads* distributed by the bus encompass a heterogeneous data ontology structured into three levels of abstraction, as detailed in Table 1.

Asynchronous events allow the graphical interface to continue rendering while analytical computations run or data are serialised. Likewise, the clinical module can process performance information without interrupting sensor acquisition or the visual *feedback* loop.

Therefore, the event bus provides both temporal and functional decoupling among the system components. It also facilitates extensibility, as new modules can be incorporated as subscribers without modifying the internal implementation of existing publishers.

### 3.4. Application Layer

The application layer contains the components directly involved in the gamified exercise and in the automated interpretation of participant performance. It is divided into two functional sublayers: the semi-automated analytics layer and the WebGL and gamification layer.

#### 3.4.1. Semi-Automated Analytics Layer

The semi-automated analytics layer contains the clinical module that processes the aggregated performance events generated during each training session. As shown in Figure 1, this module receives a specific *payload* through the web bus, composed of performance events and their corresponding timestamps. These events summarise the relevant information produced during the interaction, including task completion, recorded errors, trajectory deviations, collisions, and other indicators required for subsequent analysis.

Its purpose is to transform these session events into interpretable indicators. Depending on the exercise configuration, the module calculates temporal metrics, such as total duration and cumulative time spent outside the trajectory; spatial metrics, such as the number of deviations or collisions; and overall performance variables, such as movement continuity, completion status, and the score obtained in the gamified task.

The module also automatically identifies potentially relevant events by assigning markers or *flags*. These may indicate a high number of errors, prolonged time spent outside the trajectory, exercise interruptions, incomplete data, or performance patterns requiring professional review.

The term “semi-automated” emphasises that the module does not generate an autonomous clinical diagnosis. It performs initial computational screening and summarises session data, but a qualified professional must review the resulting metrics and markers.

To avoid interfering with the real-time interaction loop, analytical processing runs asynchronously. Data may be grouped into batches (*batching*) before serialisation or storage, thereby reducing the number of individual write operations and limiting computational load during the exercise.

#### 3.4.2. WebGL and Gamification Layer

The WebGL and gamification sublayer includes the web interface module and the evaluation module. Both are decoupled through the event bus but consume different *payloads*. The web interface module primarily receives the estimated orientation, expressed as quaternions or Euler angles. The evaluation module receives XYZ vectors and events related to trajectory deviations and errors detected during task execution.

The web interface module is implemented using web technologies and the Three.js graphics library. Its main function is to translate the participant’s head orientation into cursor, object, or virtual-representation movement within the exercise environment. It receives this information through orientation events published on the bus and uses it to update the cursor or virtual object associated with the participant’s head movement.

The browser rendering loop updates the graphical scene, targeting an update rate of approximately 60 frames per second. In each rendering cycle, the system uses the most recent available orientation state. This approach allows sensor updates and graphical rendering to operate at related but logically independent frequencies.

**Figure 4:**
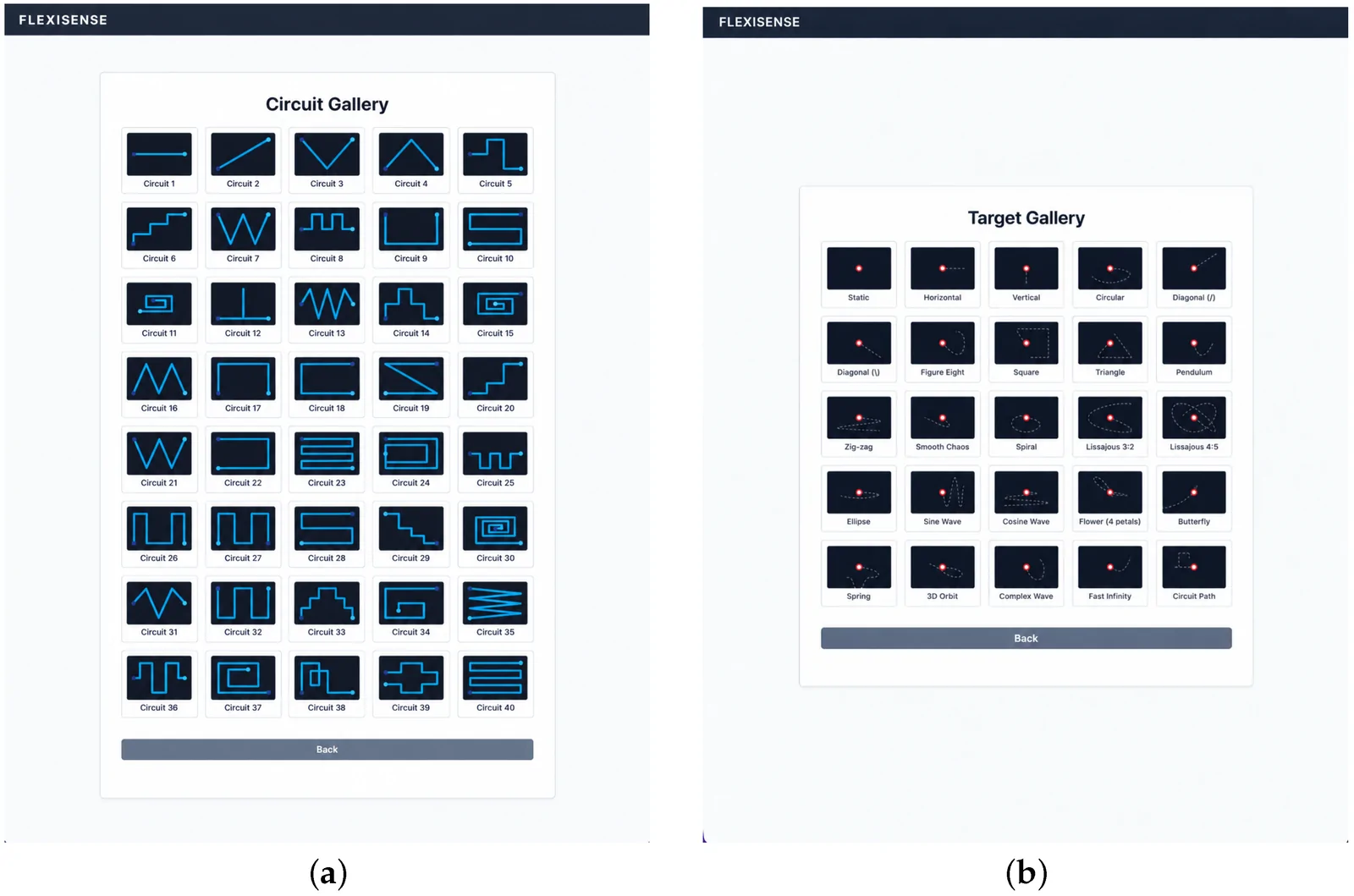
Galleries of exercise modalities available in the system. (**a**) Circuit gallery, presenting predefined 2D geometric trajectories of varying complexity for head-orientation tracking training. (**b**) Target gallery, presenting dynamic continuous moving target patterns.

The platform provides a diverse library of gamified exercises structured into two main modalities, illustrated in Figure 4: a circuit gallery containing 2D geometric paths of varying complexity, and a target gallery containing dynamic continuous moving target patterns.

**Figure 5:**
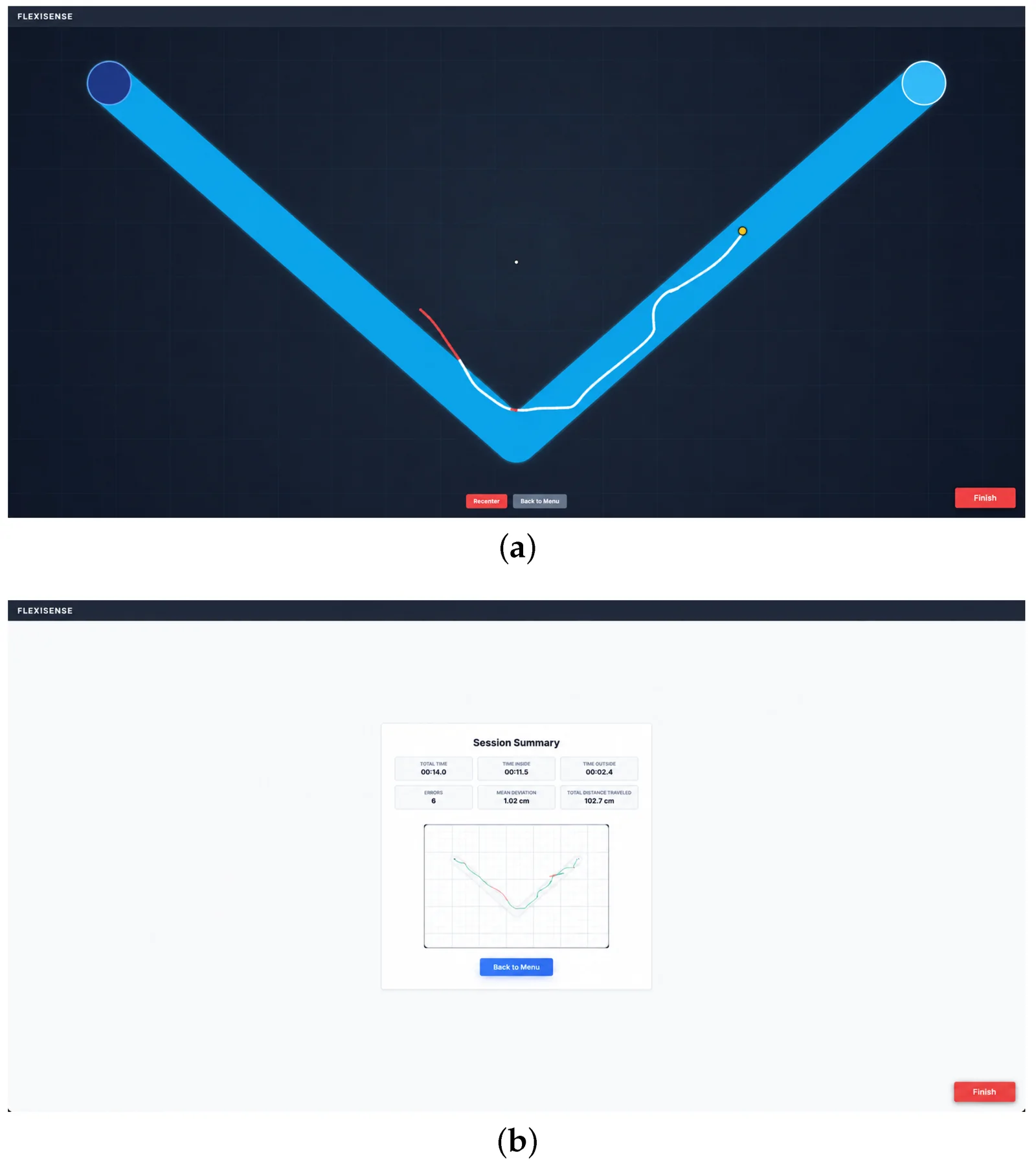
Web application interface of the gamified cervical training system. (**a**) Real-time task execution screen, illustrating the prescribed V-shaped trajectory corridor, the cursor steered via IMU head orientation, visual deviation feedback (red trail segment), and live performance indicators in the top bar. (**b**) Post-session summary screen, displaying aggregated performance metrics (total time, time inside/outside corridor, error count, mean deviation, and total distance travelled) alongside the reconstructed trajectory graph.

During task execution, the interface displays the prescribed trajectory corridor, the cursor steered in real time by head orientation, live performance timers and metrics in the upper navigation bar, and immediate visual feedback when departing from the permitted path (Figure 5a). Upon task completion, the interface generates a session summary card (Figure 5b) presenting aggregate metrics (total execution time, time within and outside the corridor, error count, mean spatial deviation, and total distance travelled) along with a graphical reconstruction of the recorded trajectory highlighting compliant tracking in green and deviations in red.

The evaluation module implements the spatial logic required to assess task execution. Through the event bus, it receives the XYZ vectors representing the spatial state of the interactive elements, together with trajectory-related events. Using this information, it compares the current position of the participant-controlled element with the geometric boundaries of the prescribed trajectory.

Collision and deviation detection uses Axis-Aligned Bounding Boxes (AABB). The AABB method defines rectangular spatial boundaries around relevant virtual objects or trajectory regions. During each evaluation cycle, the module determines whether the participant-controlled element intersects a valid target region or leaves the permitted movement corridor.

When the module detects a collision or deviation from the prescribed trajectory, it generates an error event that includes the incident type, timestamp, spatial position, and, where applicable, the magnitude or duration of the deviation. These events are then republished to the bus so the clinical module can incorporate them into the aggregated performance events it consumes. In parallel, the WebGL interface uses the evaluation state to generate immediate visual or auditory *feedback*.

This separation between graphical rendering and logical evaluation prevents the interface code from simultaneously assuming responsibility for visualisation and clinical scoring. It also lets evaluation rules be modified without redesigning the graphical environment.

### 3.5. Portal and Management Layer

The portal and management layer provides the interface used by the physiotherapist or supervising professional. It receives the processed metrics and markers (*flags*) generated by the clinical module and presents them through a web-based dashboard.

The portal supports session review and longitudinal monitoring. It can display completed exercises, execution times, error counts, time spent outside the trajectory, calculated scores, flagged events, and changes across consecutive sessions.

The management layer also allows remote configuration of session parameters, including difficulty level, target trajectory, tolerance limits, exercise duration, *feedback* modalities, and number of repetitions. As represented in Figure 1 by the flow labelled Exercise Configuration, these decisions are transferred to the participant’s session and determine the conditions under which the subsequent interaction is performed.

The portal operates outside the immediate kinematic-processing loop. Its main functions are metric review, longitudinal monitoring, session configuration, and decision support. In this way, remote operations do not interfere with acquisition, *edge* processing, or real-time *feedback* generation.

### 3.6. System Operation: Closed-Loop Interaction, Professional Supervision, and Extensibility

The layers described above operate as a closed perception–action loop. The participant performs a head movement captured by the IMU accelerometer and gyroscope; the data are transmitted via BLE to the *edge* layer, where they are filtered and fused to estimate head orientation. The system publishes the result to the event bus, which delivers the orientation to the WebGL interface module and the spatial vectors and trajectory events to the evaluation module. The interface updates the virtual environment while the evaluator checks whether the movement remains within the prescribed boundaries and, when it detects a deviation, generates immediate visual or auditory *feedback*. The participant perceives this cue and corrects the movement, which the sensor then captures again, closing the loop. Consequently, the flow can be summarised as follows: head movement → inertial acquisition → BLE transmission → filtering and sensor fusion → event publication → graphical update and trajectory evaluation → audiovisual *feedback*→ movement correction.

In parallel, the bus delivers performance events and their timestamps to the clinical module, which calculates scores and automatic markers and then provides them to the portal. This separation keeps the time-critical components (acquisition, orientation estimation, evaluation, and graphical feedback) within the local processing environment, while aggregated analysis and clinical review are performed outside the interactive loop.

The *human-in-the-loop* supervision represented in Figure 1 operates on this aggregated information. The clinical module summarises large volumes of movement and interaction data but does not generate an autonomous diagnosis: the professional interprets the metrics and markers on the dashboard, considers them alongside the participant’s clinical and functional context, and decides whether to maintain or adjust the exercise configuration for subsequent sessions. Automation therefore supports review and decision-making without replacing professional judgment.

This decoupled organisation also supports system evolution. The *wearable* sensor can be replaced with another IMU, provided its output is adapted to the expected acquisition format, and the sensor-fusion algorithm can be replaced without affecting the analytical or graphical modules. The WebGL scene and exercise logic support new trajectories, game mechanics, *feedback* modalities, or scoring procedures, while new analytical modules can subscribe to the bus to calculate more advanced indicators or apply machine learning methods to the recorded time series. The event-driven model also supports integrating additional sensors, such as a second IMU placed on the trunk, which would enable calculation of relative head-trunk motion and better isolation of cervical movement from thoracic compensations.

### 3.7. Technical Verification: Latency, Packet Loss, and Frame Rate

To evaluate the technical performance and temporal determinism of the proposed architecture, a benchtop laboratory benchmarking campaign was conducted. The experimental setup comprised the wearable sensing unit (Seeed Studio XIAO nRF52840 Sense with onboard LSM6DS3TR-C 6-DOF IMU) mounted on a static test bench at a nominal operating distance of 1.5 m line-of-sight from the host workstation (Intel Core i7, 16 GB RAM, dedicated GPU, 1920 × 1080 display at 60 Hz, Microsoft Windows 11, Google Chrome v120+, 64-bit). The IMU streamed calibrated 3-axis accelerometer and 3-axis gyroscope data at a nominal output data rate of 60 Hz (T=16.67 ms) via BLE GATT notifications. The web application continuously received inertial packets through the Web Bluetooth API, executed client-side Madgwick sensor fusion, and updated a 3D WebGL cursor along target trajectories through the asynchronous event bus, with the rendering loop targeting 60 FPS.

The benchmarking campaign comprised ten independent trials of 10 min (600 s) each under nominal tracking workload, yielding approximately 36,000 nominal data packets per trial and >360,000 packets across the campaign (>358,500 valid received packets). The browser application was instrumented using the W3C High Resolution Time API (window.performance.now()) to record monotonic timestamps with sub-millisecond precision. Device-side timestamps were generated by the nRF52840 real-time counter (32.768 kHz clock with 32-bit software extension to handle 24-bit hardware rollover) and embedded into the 20-byte BLE payload alongside a 16-bit monotonic sequence counter and inertial data. Initial clock alignment between device and host time domains was established through bidirectional round-trip GATT exchanges prior to each trial.

End-to-end latency was operationally defined as the cumulative elapsed time from IMU physical sampling to WebGL frame rendering submission. To isolate temporal contributions across the software and communication stack, timing was instrumented across four constituent stages: (i) *IMU acquisition and FIFO readout delay* (8.33±0.85 ms), quantified on the microcontroller by logging the hardware timer at the IMU Data-Ready interrupt relative to the 60 Hz internal sensor output cycle, corresponding on average to half the sampling period (T/2≈8.33 ms) plus sensor digital filtering and readout overhead; (ii) *BLE transmission and host reception delay* (14.18±2.65 ms), obtained via synchronized differential timestamping ($t_{\mathrm{rx}}-t_{\mathrm{tx}}$) between the device RTC timestamp embedded in the GATT payload and the browser reception timestamp captured at the characteristicvaluechanged event via window.performance.now(), corrected for the initial clock offset (capturing packet encapsulation, queueing, over-the-air transfer across the negotiated 7.5–15.0 ms connection interval, and host OS Bluetooth HCI driver handling); (iii) *Client-side event dispatch and Madgwick sensor fusion* (0.64±0.12 ms), measured directly within the JavaScript runtime using high-resolution performance.now() timers bookending packet parsing, internal event bus publication, and execution of the Madgwick gradient-descent quaternion update; (iv) *WebGL rendering queueing and draw execution* (8.35±1.20 ms), measured as the interval between orientation state availability and completion of the subsequent requestAnimationFrame render callback submission to the GPU, reflecting VSync alignment (averaging half the 16.67 ms refresh frame at 60 Hz) plus draw overhead. The mean end-to-end latency across the ten trials was 31.5±3.8 ms (sum of stage means: 8.33+14.18+0.64+8.35=31.50 ms, with trial-to-trial standard deviation ±3.8 ms), well below the 100 ms value commonly used as a design guideline for interactive feedback.

BLE packet loss was determined for each trial as $100\times \left(N_{\mathrm{expected}}-N_{\mathrm{received}}\right)/N_{\mathrm{expected}}$ using sequence counter continuity ($N_{\mathrm{expected}}\approx$ 36,000 per trial), yielding a mean packet loss rate of 0.42%±0.14%. Inter-arrival jitter was calculated as the standard deviation of successive notification arrival intervals ($\Delta t_{i}=t_{i}-t_{i-1}$) within each trial, averaging 2.74±0.38 ms across trials. The graphical interface maintained a stable render-submission rate of 59.85±0.32 FPS (mean ± SD across trials). All technical measurements were obtained exclusively in a benchtop laboratory setting, not during participants’ exercise sessions. These results characterise technical performance under the tested laboratory conditions; performance under ecological conditions during actual exercise sessions remains to be evaluated.

## 4. Materials and Methods: Pilot Feasibility Study

### 4.1. Study Design

A randomised pilot feasibility study with two parallel groups and pre–post measurements was conducted following the CONSORT extension for pilot and feasibility trials [37]. The primary objective was to evaluate the operational feasibility and technical safety of the proposed architecture during an intervention program in a healthy teenager population, with kinematic metrics collected as a secondary exploratory objective. Participants were allocated in a 1:1 ratio, and assessments were performed at two time points: the week before the program began (pre) and the week after it ended (post). The study followed the Declaration of Helsinki [38] and was approved by the relevant ethics committee.

**Figure 6:**
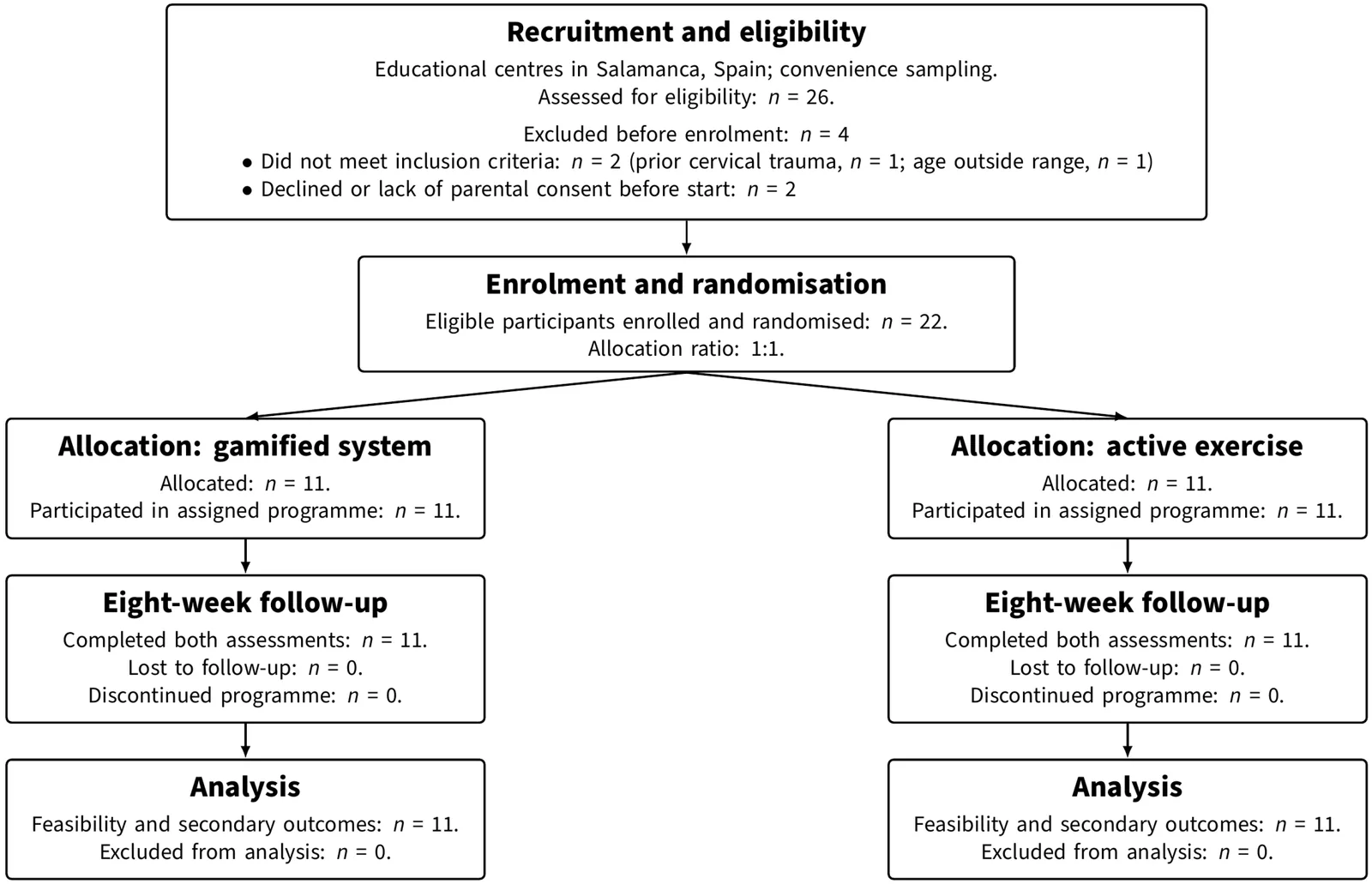
Participant flow through recruitment, enrolment, randomisation, allocation, follow-up, and analysis in the pilot feasibility trial according to the CONSORT extension for pilot and feasibility studies. Session completion was self-reported.

Figure 6 summarises participant flow through recruitment, enrolment, randomisation, allocation, follow-up, and analysis. A total of 26 potential participants were assessed for eligibility; 4 were excluded prior to randomisation (2 did not meet inclusion criteria due to prior cervical trauma or age outside the specified range, and 2 declined or did not obtain parental consent before the study began), leaving 22 eligible participants who were enrolled and randomised.

### 4.2. Participants

The sample comprised 22 healthy teenagers aged 16 to 18 years, recruited from educational centres in Salamanca (Spain) using convenience sampling. Inclusion criteria were: age between 16 and 18 years, absence of active neck pain limiting exercise performance, and availability of informed consent signed by the legal guardian, along with the participant’s assent. Participants were excluded if they had a history of cervical or craniocerebral trauma, diagnosed vestibular or neurological disorders, previous cervical spine surgery, uncorrected visual impairments that prevented them from following the on-screen target, or concurrent physiotherapy treatment for the cervical region.

Participants were randomly allocated to two groups (experimental and control) using a centralised random-number generator with allocation concealment. Consistent with the exploratory nature of the study design, no formal sample size calculation was performed to detect between-group efficacy differences. The sample size was determined based on feasibility criteria and participant availability, in line with common recommendations for pilot studies, whose purpose is to estimate feasibility parameters and variability in order to inform the design and sample size of subsequent confirmatory trials.

### 4.3. Study Variables

#### 4.3.1. Primary Variables (Feasibility, Adherence, and Safety)

To determine the operational feasibility of the technological ecosystem, the following variables were monitored:

- **Adherence rate:** Percentage of completed sessions relative to the total number prescribed, expressed as absolute numerator and denominator values. Adherence was documented through self-report using a written tracking sheet completed by each participant. The sessions were not directly supervised, and adherence was not automatically recorded through the portal during this study phase.
- **Technical and safety events:** Recording of any BLE connection failure, interface desynchronization, or occurrence of adverse events (e.g., dizziness, cybersickness, or increased neck pain), as reported by participants and verified during the in-person assessment sessions.

Following recommendations for pilot studies, the following feasibility success thresholds were established a priori: (i) a mean adherence rate ≥80% of the prescribed sessions, (ii) a retention rate ≥80% of randomized participants, (iii) absence of serious adverse events attributable to the intervention, and (iv) absence of technical failures preventing completion of a session in more than 10% of the sessions performed. Simultaneous fulfilment of all four criteria was defined as the condition for considering progression to a larger-scale controlled trial feasible.

#### 4.3.2. Exploratory Secondary Variables (Kinematics)

Head-orientation tracking performance was assessed exploratorily using a standardised circuit task with the modular system described above. The assessment was standardised in a seated position, whereas the training sessions were performed in standing (Section 4.4.1). This choice was deliberate and based on two criteria. First, sitting with arm support restricts the degrees of freedom of the trunk and lower limbs, thereby reducing postural variability between participants and between the pre- and post-assessment time points, which is particularly important when measuring small-magnitude angular errors. Second, it standardises the assessment posture with that required for the HRA test, allowing both measurement blocks to be collected under a single condition. In contrast, standing training was intended to impose a greater demand on overall postural integration. As a limitation inherent to a feasibility setting, measurements were obtained using a single frontal IMU without thoracic stabilisation straps. Therefore, the metrics capture combined head movement together with any possible trunk compensations.

The following kinematic outcome variables were recorded:

- **Circuit completion time (seconds):** Total time required to complete the trajectory.
- **Number of errors:** Total number of deviations from the predefined trajectory.
- **Time outside the trajectory (seconds):** Total duration for which the cursor remained outside the prescribed path.

Higher values for these variables were interpreted as indicating poorer head-orientation tracking performance.

#### 4.3.3. Pain Intensity

Pain intensity was assessed using a Visual Analog Scale (VAS), recorded before and after the eight-week intervention. A score of 0 indicated the absence of pain, whereas a score of 10 represented the maximum imaginable pain. Participants rated their pain at the time of assessment according to this scale.

#### 4.3.4. Head Repositioning Accuracy (HRA)

Head repositioning accuracy was assessed using the Head Repositioning Accuracy (HRA) test, following procedures previously described in the literature [39,40]. Participants sat with their arms supported and their eyes closed to minimise visual input and primarily assess cervical proprioception.

Initially, the examiner passively guided the participant’s head to a predefined target position for each of the following movements: flexion, extension, left rotation, and right rotation. The examiner held each position briefly to allow sensory encoding of the movement, and performed three familiarisation trials for each direction.

Next, participants actively reproduced the previously attained position starting from the neutral position. Repositioning error was recorded as the angular difference, in degrees, between the target position and the position reproduced by the participant, measured with a goniometer. The assessor performing the HRA assessment was blinded to participants’ group allocation. Higher values indicate lower accuracy of cervical motor control.

### 4.4. Procedure

#### 4.4.1. Intervention Procedure (Experimental Group)

This protocol was designed to stimulate active head-orientation control through dynamic tracking tasks performed across different planes of movement.

Participants allocated to the intervention group completed an eight-week training program using the gamified system. Participants performed the intervention three times per week on non-consecutive days.

Each session comprised tracking tasks involving a moving target, set to difficulty level 3 on the device. Participants were required to guide a cursor through controlled head movements in order to follow a continuously moving target as accurately as possible.

Participants performed training standing to promote greater overall postural integration, positioned approximately 2 m from the screen.

Each repetition lasted 30 s. Three repetitions were performed for each type of trajectory, with an approximate 20 s rest period between repetitions. The difficulty level remained constant throughout the intervention to ensure a homogeneous training stimulus across participants.

The program included different target movement patterns, always performed in the same order to ensure protocol standardisation: (a) horizontal trajectories; (b) vertical trajectories; (c) circular trajectories.

The sessions were not directly supervised. Participants recorded completion of each session on a written tracking sheet designed to monitor adherence to the program. Adequate adherence was defined as completion of at least 80% of the scheduled sessions.

#### 4.4.2. Intervention Procedure (Control Group)

This program represented a conventional intervention based on active cervical mobility exercises, without a specific sensorimotor training component.

Participants allocated to the control group completed an active cervical exercise program over eight weeks, with three sessions per week on non-consecutive days, matching the intervention group’s session frequency. Participants performed each session standing under the same conditions as the experimental group.

The program consisted of active cervical mobility exercises performed across different planes of movement, including: (a) cervical flexion–extension movements; (b) cervical rotations; (c) lateral flexion movements; (d) combined movements consisting of tracing the participant’s name using head movements.

For each exercise type, participants performed 10 repetitions, with an approximate 20 s rest period between movement blocks. Participants always performed the exercises in the same order to standardise the protocol.

As in the experimental group, the sessions were not directly supervised. Participants recorded session completion on a tracking sheet designed to monitor adherence to the program. Adequate adherence was defined as completion of at least 80% of the scheduled sessions.

### 4.5. Statistical Analysis

IBM SPSS Statistics (version 30, IBM Corp., Armonk, NY, USA) was used for statistical analyses. Variable normality was assessed using the Shapiro–Wilk test. As most variables did not follow a normal distribution (*p* < 0.05), non-parametric tests were used for inferential analysis.

Descriptive analyses are reported as medians and first-third quartiles. For within-group analysis (pre–post), the Wilcoxon signed-rank test was used to assess changes within each group after the intervention. Subsequently, differences between the pre- and post-intervention measurements were calculated for each variable and used for between-group analysis with the Mann–Whitney U test for independent samples.

In addition, effect sizes were calculated using the *r* statistic derived from the standardised *Z* value of each non-parametric test. For within-group comparisons (Wilcoxon signed-rank test), $r=\left|Z\right|/\sqrt{n}$ was used, where *n* is the number of paired observations analysed in the corresponding group (n=11). For between-group comparisons (Mann–Whitney U test), $r=\left|Z\right|/\sqrt{N}$ was used, where *N* is the total number of participants included in the comparison (N=22). Effect sizes were interpreted as small (0.10), moderate (0.30), and large (0.50).

The level of statistical significance was set at p<0.05. Given the pilot and exploratory nature of the study, no formal adjustment for multiple comparisons was applied. Testing multiple outcomes without correction increases the risk of Type I error (false-positive findings). Secondary analyses were intended to explore preliminary patterns and estimate potential effects rather than to provide confirmatory evidence of efficacy; therefore, the resulting *p*-values should be interpreted cautiously and descriptively. For between-group comparisons of pre–post changes, Hodges–Lehmann estimates with 95% confidence intervals were additionally calculated to provide information on the magnitude and precision of the observed differences.

Regarding data handling, the analysis followed an intention-to-treat approach, with each participant analysed in the group to which they were initially assigned, regardless of adherence. As there were no dropouts or losses to follow-up and complete pre- and post-intervention measurements were available for all 22 participants, no missing-data imputation procedures were required.

Given the exploratory pilot design and the small sample size, comparative results (particularly the absence of statistically significant between-group differences) should not be interpreted as formal evidence of efficacy, equivalence, or non-inferiority, but rather as preliminary estimates to inform the design of future trials.

## 5. Results

### 5.1. Sample Characteristics

A total of 22 participants were analysed, distributed into two groups of 11 participants each. No losses to follow-up or dropouts were recorded; therefore, all 22 randomised participants completed the entire eight-week program and both assessments (pre and post). Consequently, the intention-to-treat analysis matched the per-protocol analysis. Baseline demographic characteristics of the participants are presented in Table 2. The groups were comparable with respect to the variables reported in Table 3, with no statistically significant between-group differences in any of them before the intervention (p>0.05).

### 5.2. Operational Feasibility and Safety (Primary Variables)

Regarding the primary feasibility metrics, no participant-reported connectivity or *hardware* events prevented session completion or required a repeat. The self-reported adherence rate was 100% of the planned sessions (528/528 scheduled sessions: 24 sessions per participant × 22 participants). This figure was based on participant-completed tracking sheets and therefore represents a self-reported estimate rather than an objectively verified adherence measure. Likewise, retention was 100% (22/22 randomised participants completed the program and both assessments). Based on these participant-reported data, the prespecified feasibility thresholds were assessed as fulfilled, although adherence and technical events were not objectively verified through automatic system logging. No adverse events (e.g., dizziness, cybersickness, or increased neck pain) were recorded during the intervention or assessments. No participant-reported graphical-interface desynchronization events or BLE pairing failures prevented completion of the prescribed exercise. Separately, technical measurements were obtained exclusively in a benchtop laboratory setting (Section 3.7): end-to-end latency was 31.5±3.8 ms, inter-arrival jitter was 2.74±0.38 ms, BLE packet loss was 0.42%±0.14%, and frame rate was 59.85±0.32 FPS. These metrics were not measured during participants’ actual exercise sessions and should not be interpreted as measurements of in-session performance. Performance under ecological conditions remains to be evaluated.

### 5.3. Exploratory Kinematic Results (Secondary Variables)

For the exploratory secondary metrics, descriptive analysis of median values (Table 3) showed a clear post-intervention reduction in temporal performance metrics (total completion time and time outside the trajectory) in both groups. For the remaining variables (errors, pain, and HRA), the median values tended to remain stable or showed only marginal reductions, suggesting a possible floor effect. No statistically significant between-group differences were found in pre–post changes for any of the variables analysed (*p* > 0.05).

In the experimental group, total completion time (Z = −2.135; *p* = 0.033; r = 0.64) and time outside the trajectory (Z = −2.937; *p* = 0.003; r = 0.89) improved significantly, with large effect sizes for both variables.

In the control group, total completion time improved significantly (Z = −2.845; *p* = 0.004; r = 0.86), whereas the reduction in time outside the trajectory did not reach statistical significance in this group (p>0.05).

No statistically significant changes were observed in the remaining variables (errors, pain, and the four directions of the HRA test) in either group (*p* > 0.05).

The between-group effect size was moderate for total completion time (r = 0.34) and small for the remaining variables (r < 0.30).

The between-group comparisons of pre–post differences are detailed in Table 4. None of the eight between-group comparisons reached statistical significance, and effect sizes ranged from small to moderate. With 11 participants per group and no formal sample size calculation for efficacy, these findings do not establish equivalence or exclude potentially relevant between-group differences.

## 6. Discussion

The primary objective of this study was to design, implement, and evaluate the operational feasibility of a modular and gamified technological architecture for training head-orientation tasks.

The main technological contribution of this study is the platform’s modular organisation. The design separates data acquisition from the *wearable* sensor, signal fusion, and WebGL interface rendering. This separation is intended to reduce interference between tasks during the perception–action cycle. In this pilot, no participant-reported connectivity or system-related event prevented session completion. The architecture enables replacing the inertial hardware or updating the graphics engine without rewriting the evaluation logic. The management portal allows professionals to review aggregated session information asynchronously. The benchtop measurements reported in Section 3.7 provide an initial characterisation of the platform under laboratory conditions, with an end-to-end latency of 31.5±3.8 ms and a frame rate of 59.85±0.32 FPS. They do not establish performance during actual exercise sessions or demonstrate an advantage over alternative architectures. Technical performance under ecological conditions remains to be evaluated.

The clinical results should be interpreted as feasibility data. Self-reported adherence was 100%, and no adverse events were reported, including cybersickness or exacerbation of neck pain. However, adherence was based on participant self-report using written tracking sheets and was not objectively verified through automatic portal logging. A limitation of the present feasibility assessment is that adherence and technical events were self-reported and were not automatically logged by the system. Therefore, these measures could not be independently verified. Future studies should incorporate automatic session logging to provide objective measures of adherence and technical performance. Both groups showed exploratory reductions in task completion times. Because this pattern was observed in both study arms, it cannot be specifically attributed to audiovisual *feedback*. The literature on gamification in teenagers [25,26] supports effects on motivation and perceived effort, but this is insufficient to establish clinical efficacy in the cervical context. This pilot study provides preliminary evidence of operational feasibility. Its exploratory comparisons do not allow clinical superiority of the gamified system over the active cervical exercise programme to be established; differential efficacy requires evaluation in an adequately powered trial designed for that purpose.

With this in mind, the randomised trials and systematic reviews available in this field evaluate commercial immersive virtual reality or head-mounted displays in adults with chronic neck pain, and report clinical endpoints such as pain intensity, disability and kinesiophobia [30,31,32,41,42]. None of them describes the architecture that supports the interaction loop or reports how the system performs during use. The present work addresses this gap with a single low-cost IMU, sensor fusion and rendering executed in the browser without installation, kinematic tracking metrics as the outcome, and an evaluation conducted in healthy adolescents rather than symptomatic adults. In the randomised trial closest in design to ours [32], virtual reality was not superior to an active neck exercise programme for pain intensity, range of motion, neck disability or pressure pain thresholds, and both groups improved over time. A systematic review with meta-analysis reports that virtual reality and conventional training produce similar effects on overall neck range of motion and pain intensity [31]. Meta-analyses that favour virtual reality either pool heterogeneous comparators, including no intervention and usual care [30], or report effects present immediately after the intervention but no longer detectable at follow-up [41,42]. Our comparison involved two active interventions matched in frequency, duration and posture, so the absence of a between-group difference is consistent with findings reported in this literature, and the improvement observed in the control arm is itself expected given the documented effects of active cervical exercise programmes [20,21].

Total completion time decreased significantly in both groups, and the within-group effect size was larger in the control group (r=0.86) than in the experimental group (r=0.64). This result may reflect familiarisation with the assessment circuit, since the same procedure was repeated during the pre- and post-intervention measurements. Participants could not be blinded to group allocation because of the nature of the interventions. Although the outcome assessor was blinded to group allocation, participants received three familiarization trials for each movement direction before the active HRA assessment; however, no separate familiarization session was conducted for the full assessment circuit before baseline. Therefore, a learning or familiarization effect related to the repeated assessment circuit cannot be completely excluded. The control group also received an active cervical mobility program with the same frequency, duration, and posture [20,21]; therefore, the comparison involved two active interventions. In addition, baseline pain values (median ≤1 on the VAS) and repositioning error values (medians ranging from $1^{\circ }$ to $2^{\circ }$) left little room for improvement in asymptomatic teenagers. Time outside the trajectory was the only variable that showed a statistically significant reduction in the experimental group (r=0.89) but not in the control group. This pattern appears from two separate within-group comparisons. The between-group comparison of pre–post changes did not reach statistical significance (U=39.5; Z=−1.382; p=0.167; r=0.29; Table 4). A statistically significant within-group change in one arm and a non-significant change in the other do not establish a difference between the interventions. Because multiple outcomes were tested without correction, the risk of Type I error is increased; the isolated significant result for time outside the trajectory in the experimental group should therefore be interpreted with particular caution. Taken together, improvements in both groups and the absence of a significant between-group difference are compatible with familiarisation with the assessment task, possible floor effects in outcomes with low baseline values, and limited statistical power. These explanations cannot be disentangled by this pilot study. The absence of a statistically significant between-group difference establishes neither differential efficacy nor equivalence between the interventions.

## 7. Conclusions

This study presents a modular event-driven platform for recording head orientation and using it in gamified head-orientation training tasks. The platform combines a wearable IMU, local signal processing, and a WebGL interface, while the management portal enables the supervising professional to review training sessions asynchronously. The decoupled layered organisation allows the inertial hardware to be replaced or the graphics engine to be updated without rewriting the evaluation logic.

The pilot evaluation in healthy teenagers provided preliminary evidence of operational feasibility under the tested conditions. During the eight-week intervention, no participant-reported technical events prevented session completion, self-reported adherence and retention were 100%, and no adverse events were reported. Given the self-reported nature of technical events and adherence, these findings provide preliminary support for progression to a larger-scale controlled trial with objective automatic session logging.

The kinematic metrics were collected for exploratory purposes. Both groups showed reductions in task completion time, whereas the reduction in time outside the trajectory was statistically significant only in the experimental group, while the between-group comparison of pre–post changes did not reach statistical significance. Improvements in both groups, together with the absence of a significant between-group difference, are compatible with familiarisation, possible floor effects in outcomes with low baseline values, and limited statistical power. The isolated within-group finding for time outside the trajectory warrants caution because the analyses were not corrected for multiple comparisons, increasing the risk of Type I error. These data provide preliminary estimates to inform future studies. The exploratory comparisons do not allow clinical superiority to be established; differential efficacy remains to be evaluated in an adequately powered trial designed for that purpose.

The scope of this pilot should be taken into account when interpreting its results. The sample comprised healthy teenagers without active neck pain limiting exercise performance, consistent with the eligibility criteria for this initial assessment of operational feasibility and safety. This choice limits what the exploratory outcomes can show, where the baseline values were close to the floor of their respective scales, with median pain of 1 and 0 points on the VAS in the experimental and control groups and median repositioning errors between $1^{\circ }$ and $2^{\circ }$ in all four directions of the HRA test. These low baseline values may have limited the scope for detecting improvement. Together with the small sample and limited statistical power, they complicate the interpretation of non-significant between-group differences; the absence of such differences cannot be attributed conclusively to the asymptomatic population. A second constraint concerns what the sensor configuration measures. A single head-mounted IMU without thoracic fixation estimates head orientation relative to space, so trunk movement enters the signal and cannot be split from cervical motion. Assessment was conducted in a seated position with the arms supported, which restricts trunk and lower-limb degrees of freedom without immobilising the torso. The same posture was used at both time points, so any systematic compensation affects the within-participant comparison only to the extent that it changed between measurements. Neither of these conditions isolates cervical motion, and the kinematic variables recorded by the system should be read as head-orientation tracking performance. The Head Repositioning Accuracy test is not affected by this constraint, since it is measured with a goniometer by an examiner and retains its usual interpretation as a proprioceptive measure.

Therefore, the findings obtained in this healthy, asymptomatic population should not be directly extrapolated to symptomatic populations. Future controlled trials should evaluate the system in participants with neck pain or impaired cervical motor control. In such trials, change in Head Repositioning Accuracy (HRA) repositioning error could be considered as the primary outcome, with pain and functional disability assessed as secondary clinical outcomes.

Within the V3 framework [15], this work addresses system verification and provides an initial feasibility assessment. Metric characterisation against an optoelectronic reference system is the natural next step, along with incorporating a second thoracic IMU to better isolate cervical movement from trunk compensations. Further research directions include evaluation in symptomatic populations, automatic session logging through the portal itself, support for telerehabilitation with asynchronous monitoring, and analysis of movement patterns using machine learning models.

## Figures and Tables

**Table 1:** Functional classification of the *payloads* distributed by the event bus according to the consuming module.

Consuming Module	Event Category	Payload Content
Clinical Module	Performance events	Aggregated performance events and timestamps required for the preliminary analysis of the session.
Web Interface Module	Orientation state	Estimated orientation expressed as quaternions and/or Euler angles used to update the graphical environment.
Evaluation Module	Spatial state	XYZ vectors used to determine the position of the interactive elements.
Evaluation Module	Trajectory and error events	Deviations from the prescribed trajectory, collisions, error events and associated data.

**Table 2:** Baseline characteristics of the participants.

Characteristic	Intervention (n = 11)	Control (n = 11)
Age (years)	17.36±0.50	17.27±0.47
Female, n (%)	7 (63.60%)	6 (54.50%)
Male, n (%)	4 (36.40%)	5 (45.50%)
Height (cm)	165.00±5.97	165.91±7.30
Weight (kg)	62.45±7.19	64.09±8.24

Values are presented as mean ± standard deviation (SD) or n (%). Continuous summaries and percentages are displayed to two decimal places; participant counts are integers.

**Table 3:** Descriptive results by group and assessment time point. Values are expressed as median (first–third quartile).

Variable	Time Point	Intervention Median (Q1–Q3)	Control Median (Q1–Q3)
**Total time (s)**	Pre	18.00 (12.70–22.70)	19.50 (18.00–22.20)
**Total time (s)**	Post	14.00 (11.00–16.00)	13.00 (9.20–15.00)
**Time outside trajectory (s)**	Pre	1.80 (0.50–2.80)	0.70 (0.30–1.70)
**Time outside trajectory (s)**	Post	0.30 (0.10–1.00)	0.20 (0.10–0.40)
**Errors (n)**	Pre	4.00 (2.00–6.00)	2.00 (1.00–4.00)
**Errors (n)**	Post	2.00 (1.00–4.00)	2.00 (1.00–5.00)
**Pain (VAS, 0–10)**	Pre	1.00 (1.00–2.00)	0.00 (0.00–3.00)
**Pain (VAS, 0–10)**	Post	1.00 (0.00–2.00)	1.00 (0.00–2.00)
**HRA flexion (°)**	Pre	2.00 (1.00–4.00)	1.00 (1.00–3.00)
**HRA flexion (°)**	Post	1.00 (1.00–2.00)	1.00 (1.00–2.00)
**HRA extension (°)**	Pre	2.00 (1.00–3.00)	2.00 (1.00–3.00)
**HRA extension (°)**	Post	2.00 (1.00–2.00)	1.00 (1.00–3.00)
**HRA left rotation (°)**	Pre	1.00 (1.00–3.00)	2.00 (1.00–3.00)
**HRA left rotation (°)**	Post	1.00 (1.00–3.00)	2.00 (1.00–2.00)
**HRA right rotation (°)**	Pre	1.00 (1.00–2.00)	2.00 (1.00–2.00)
**HRA right rotation (°)**	Post	1.00 (0.00–2.00)	1.00 (1.00–2.00)

s: seconds; °: degrees; n: count; VAS: visual analogue scale (points, 0–10); HRA: Head Repositioning Accuracy. All medians and quartiles are displayed to two decimal places.

**Table 4:** Between-group comparisons of pre–post differences (Mann–Whitney U test) and Hodges–Lehmann estimates with 95% confidence intervals.

Variable	U	Z	*p*	r	HL Estimate (95% CI)
Total time (s)	36.50	−1.577	0.115	0.34	2.00 (−1.00 to 6.70)
Time outside trajectory (s)	39.50	−1.382	0.167	0.29	−0.50 (−1.90 to 0.20)
Errors (n)	41.50	−1.269	0.204	0.27	−1.00 (−3.00 to 1.00)
Pain (VAS, 0–10)	59.00	−0.103	0.918	0.02	0.00 (−2.00 to 1.00)
HRA flexion (°)	54.50	−0.421	0.674	0.09	0.00 (−1.00 to 1.00)
HRA extension (°)	54.00	−0.450	0.653	0.10	0.00 (−1.00 to 1.00)
HRA left rotation (°)	54.00	−0.421	0.674	0.09	0.00 (−1.00 to 1.00)
HRA right rotation (°)	57.00	−0.244	0.807	0.05	0.00 (−1.00 to 1.00)

Note: HL: Hodges–Lehmann estimate of the between-group difference in pre–post changes; CI: confidence interval. Estimates and confidence limits use the units of the corresponding variable (s: seconds; °: degrees; n: count; VAS: visual analogue scale points). Estimates, confidence limits, *U*, and *r* are displayed to two decimal places; *Z* and *p* are displayed to three decimal places. Test statistics, *p*-values, and effect sizes are dimensionless.

## Data Availability

The data presented in this study are available on request from the corresponding author. The data are not publicly available because they were collected from minor participants and are subject to privacy restrictions.
